# *In vivo* Evaluation of Fibrous Collagen Dura Substitutes

**DOI:** 10.3389/fbioe.2021.628129

**Published:** 2021-02-18

**Authors:** Wenbo Liu, Xin Wang, Jinlei Su, Qingsong Jiang, Jing Wang, Yang Xu, Yudong Zheng, Zhihui Zhong, Hai Lin

**Affiliations:** ^1^School of Material Science and Engineering, University of Science and Technology Beijing, Beijing, China; ^2^Laboratory of Nonhuman Primate Disease Modeling Research, State Key Laboratory of Biotherapy, West China Hospital, Sichuan University, Chengdu, China; ^3^National Engineering Research Center for Biomaterials, Sichuan University, Chengdu, China

**Keywords:** collagen, dura substitute, animal study, preclinical (*in vivo*) studies, safety and efficacy

## Abstract

Dura substitutes are applied in duraplasty to repair lost or damaged dura. Collagen-based dura substitutes are mainstream products in both the US and Chinese markets. In this study, dura substitute devices with potential dura regeneration ability are evaluated. The dura substitutes are composed of fibrous type I collagen that were purified from bovine tendon. Physical and chemical characterization demonstrated that the tested dura substitute has desirable porous scaffolding structures and is composed of highly purified type I collagen. The collagen dura substitutes were further investigated *in vivo* with a rabbit model for 6 months to evaluate their safety and performance to repair and regenerate dura. No inflammation or infection was observed during the course of *in vivo* study. The integration of the collagen dura substitutes with surrounding tissue was normal as compared to native tissue. The macroscopic and microscopic histological assessments of the sampled animal tissue showed that the damaged dura were regenerated. The collagen dura substitutes were resorbed between 3 and 6 months along with newly regenerated dura. Both tissue adhesion and dura repair was the worst in blank control group as compared to those in the collagen dura substitutes. Taken together, regenerative collagen dura substitutes demonstrated with suitable physicochemical properties. The *in vivo* evaluation in a rabbit model further demonstrated the safety and performance of such substitutes for dura repair and regeneration.

## Introduction

As an important tissue of central nerve system, dura mater works as natural protective barrier of brain tissue (Adeeb et al., 2012; Zwirner et al., 2019). Defects or damages of dura mater may be caused by trauma, inflammatory or neoplastic processes, or surgical procedures, which include but not limited to standard operation procedures of craniotomy in treatment of cranial injury, and thoroughly resection of cancer-related dura mater (Bartosz and Vasterling, 1994). The incomplete dura mater could not protect brain tissue, leading to complications such as cerebrospinal fluid (CSF) leakage, intracranial infection, encephalocele, tissue adhesion, and epilepsy. Therefore, the need to close dura defects has promoted a quest for the ideal dura substitute (Wang and Ao, 2019; Go et al., 2020).

According to the guidance documents issued by the National Medical Products Administration of China (NMPA) (NMPA, 2020) and U.S. Food and Drug Administration (FDA) (U. S. Food and Drug Administration, 2000), the requirements for a dura substitute should include the following attributes: biocompatibility without induction of an immune or inflammatory response; controlled risks of infection or disease; appropriate mechanical properties, resistant to tear, and anti-leakage of cerebrospinal fluid. Dura substitutes made of non-degradable materials should be bio-inert and could replace the damaged dura permanently (Nagata et al., 1999; Ström et al., 2011; Matsumoto et al., 2013). Dura substitutes made of degradable materials should have suitable degradation properties with micro-environment for tissue remodeling and possible regeneration (Costantino et al., 2000; Yamada et al., 2002; Wang and Ao, 2019). Such degradable substitutes with appropriate pore size and porosity could be resorbed matching with the development of new tissue (Neulen et al., 2011; Sandoval-Sanchez et al., 2012). In addition, the abundance and availability of raw materials for dura substitutes would reduce the cost of the device and be economically attractive. Finally, easy-handling characteristics, convenient storage conditions and stable shelf life would be other important criteria for the dura substitute devices.

In history, autogenous membrane tissue was applied as dura substitute, which caused damage of donor sites and had limitation of size and shape (Dufrane et al., 2002; Tachibana et al., 2002; Sabatino et al., 2014; Morales-Avalos et al., 2017). Membrane-like allografts from donators was used to overcome some shortcomings of autografts (Parízek et al., 1997; Azzam et al., 2018; Turchan et al., 2018). However, the allograft was not widely applied in clinic owing to the lack of donor resources, and concerns over tissue quality, ethics, and immunogenic diseases. With the development of material science and medical technologies in recent decades, a series of dura substitutes based on synthetic polymers and nature-originated materials were developed and tested to investigate their potential in clinical applications (Pogorielov et al., 2017; Vecera et al., 2018). Some of the above devices were successfully commercialized (Schmalz et al., 2018).

Based on a search by November 10th, 2020 *via* public databases of NMPA and FDA, 14 dura substitutes from 11 companies were approved by NMPA as class III devices. 41 devices from 16 companies were cleared by FDA as 510(k) devices. The dura substitute devices and their associated materials are listed in Supplementary Table 1.

Based on the material properties and their interactions with host tissue, dura substitutes could be classified to degradable and non-degradable devices that are composed of synthetic or nature-originated materials. The non-degradable dura substitutes physically set barrier for brain tissue (including piamater, arachnoid membrane) from other tissues and prevented the leakage of cerebrospinal fluid. The degradable dura substitute gradually degraded and absorbed in host after the implantation (Zerris et al., 2007; Jing et al., 2020). For degradable dura substitutes with potential regeneration properties, during the degrading period, the endogenous cells would migrate and proliferate in the regenerative device with porous scaffolding structures, gradually reconstruct of extracellular matrix and vascularize to form new dura mater till the final repair and regeneration of damaged or lost dura mater.

Commercialized dura substitutes in Supplementary Table 1 are made of diversified polymers which possess merits and drawbacks. Non-degradable dura substitutes made of synthetic polymers such as polyurethanes generally have preferable mechanical properties and anti-leakage performance (Wong et al., 2018), but they are recognized as foreign bodies after implantation (Maehara et al., 2020). The non-degradable, crosslinked animal-derived dura substitutes such as bovine/porcine pericardium have superior mechanical strength (Sun et al., 2018), but they may cause varying degrees of rapid or delayed immune or inflammatory response after implantation. Although biological safety like infectious pathogen and immunogenic disease is an inevitable issue for acellular matrix-based dura substitutes including porcine small intestinal submucosa and bovine dermis, many measures could be carried out to control the risk under a limited degree via virus inactivation treatments and immunogen elimination methods (Li et al., 2019).

Degradable dura substitutes made of absorbable synthetic polymer avoid exogenous virus and immune reaction (MacEwan et al., 2018; Chuan et al., 2020; Ramot et al., 2020), but they face other safety and performance issues. For example, the remaining of monomer or chemical residues could affect their biocompatibility. The design of their porous microstructure and quality control requirements could increase the difficulty of their manufacturing processes. The regulation of their degradability could determine their final results of tissue repair at an extensive extent (Hemstapat et al., 2020).

Because native dura mater is primary composed by type I collagen, the purified and reconstituted fibrous type I collagen-based dura substitute could meet the biological safety requirement and potential to regenerate the dura tissue via appropriate structure design. In the fully degradable dura substitutes based on natural materials, many products are based on type I collagen derived from different animal tissues. This predominated data suggests that the collagen-based dura substitutes are acceptable for clinical applications with satisfactory results.

Type I collagen is a fibrillar and structural protein which is expressed in almost all connective tissues. Because dura mater is composed primarily of type I collagen, a reconstituted bovine collagen-based scaffold is likely to be a suitable dura substitute candidate to repair and regenerate damaged or lost dura mater (Esposito et al., 2008, 2013; Calikoglu et al., 2019). In this study, a rabbit model was applied to evaluate the *in vivo* safety and dura repair performance of two dura substitutes, while blank was set as control. Furthermore, the feasibility and potential of dura regeneration by using the fibrous type I collagen dura substitute devices were investigated and discussed.

## Materials and Methods

### Materials

All medicine or chemicals including IgG and IgM ELISA kit (Abnova, United States, Cat: KA2017 and KA2040), pentobarbital sodium (Sigma, United States, Cat: #020M2298V), saline for injection (Kelun, Sichuan China, Cat: W116051504), iodophor disinfectant (Yijieshi, Sichuan China, Cat: 15.10.22) were used as received without further treatment unless otherwise stated.

### Collagen Dura Substitutes

Dura substitutes were acquired as commercial products. DuraPair was designated as the experiment device and DuraMax as the control of animal study are from Beijing Bonsci Technology Co., Ltd. (Beijing, China) (Nie and Zhang, 2018, 2020) and Tianxinfu Co., Ltd. (Beijing, China) (Li et al., 2013), respectively. Both devices were made of type I collagen fibrils from native bovine tendon and terminally sterilized by ethylene oxide gas. Please note that manufacturing processes are proprietary and product-specific. For both DuraPair and DuraMax, type I collagen fibrils were prepared from purified Achilles tendon followed by freeze-drying processes. Because the physico-chemical properties of DuraMax were reported previously (Xia et al., 2017), the current research studied the composition and microstructure of DuraPair before focusing on the *in vivo* study which investigated both fibrous type I collagen dura substitutes for dura repair and regeneration.

### Characterization of Dura Substitutes

#### Composition Analysis: Purity and Amino Acid Analysis

The purity of collagen dura substitute (DuraPair) was defined as the ratio of type I collagen in the product. Since type I collagen can be affinitive recognized and fully degraded by type I collagenase, the comparison on electrophoresis strips of protein samples before and after the enzyme hydrolysis will indicate the type I collagen content in the sample. Accordingly, sodium dodecyl sulfate—polyacrylamide gel electrophoresis (SDS-PAGE) was applied to measure the type I collagen purity and protein impurity in collagen dura substitute. Briefly, the solid dura substitute sample was cut into small pieces around 1 mm^3^, homogenized in 0.2 M HAc and stirred to attain a final sample solution (sample A). The 1.25 Unit/mL collagenase solution was prepared by dissolving the enzyme in 20 mmol/L NaH_2_PO_4_ (pH 7.4, contained 0.1 mmol/L CaCl_2_). The collagen dura substitute was digested in the collagenase solution at 37°C for 4h, to acquire the digestion solution (sample B). The collagenase solution itself was labeled as sample C. To understand the lower limit value of protein can be detected by this method, BSA solutions with gradient concentrations were tested in advance and the lower limit of BSA (LL_BSA_) was loaded as sample D. Meanwhile, protein molecular weight ladder and type I collagen control was served as sample E and F, respectively. All the samples were equal volume mixed with sample-loading buffer and cooked for 2 min in boiled water bath in sealed 0.5 mL centrifuge tubes. Subsequently, all the samples were loaded 20 μL in the spacer gel lanes, and further followed the standard SDS-PAGE protocol using Bio-rad Mini-Protean Tetra Cell gel electrophoresis system and dyed by coomassie brilliant blue. The concentrations of spacer and separation gels were 4 and 7%, respectively. The final acquired gel with electrophoresis strips of protein samples was imaged and further analyzed.

Amino acid analysis was applied to understand the amino acid components of collagen dura substitute and estimate the proportions of different amino acids as well. Around 40 mg collagen dura substitute was carefully and accurately weighed, and fully degraded in 6 mol/L HCl at 110°C for 22 h. The dried degraded outcome was dissolved to a quantified volume, and further analyzed by Hitachi L-8900 automatic amino acid analyzer.

#### Structural Analysis: Morphology and Porosity

The morphology of the collagen dura substitute was observed by scanning electron microscopy (SEM), and the porosity was further estimated. Sample was first frozen in liquid nitrogen and the brittle fracture surface was coated with an ultrathin layer of Au/Pt in anion sputtering, and further observed by Hitachi S-4800 scanning electron microscopy.

### *In vivo* Animal Study

The animal study was approved by the Animal Care and Use Committee of Sichuan University (IACUC-2016-R-001). The animal study is complied with General Considerations for Animal Studies for Medical Device (FDA, United States) and Guidance Document for Dura Substitute Devices; Guidance for Industry (FDA, United States).

#### Surgical Procedure

**FIGURE 1:**
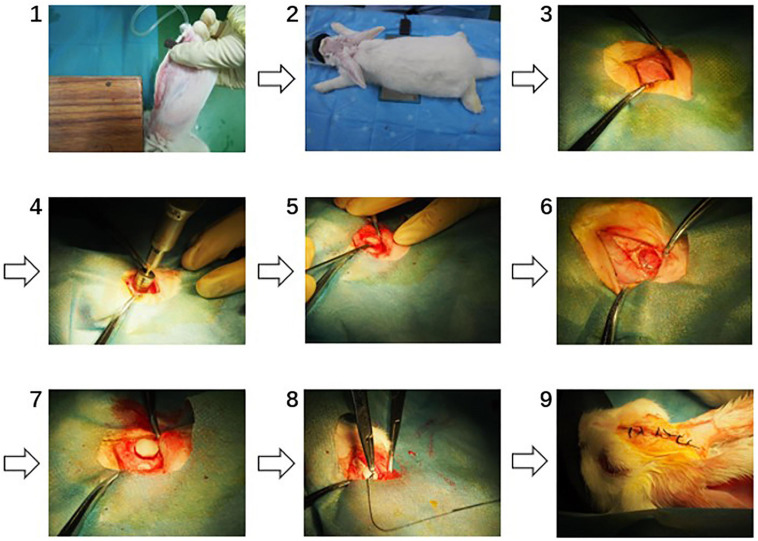
The surgical procedures of dura mater implantation (1: anaesthetization, 2: exposure of surgical site, 3: exposure of the skull, 4: round opening on the skull, 5: establishing of dura mater defect, 6: established dura mater defect, 7: dura substitute implantation, 8: suturing, 9: post-operation).

Thirty-six female SPF New Zealand white rabbits (Animal Farm of Sichuan Laboratory Animal Committee, SCXK 2013-14) weighing 2.0–3.0 kg ± 20% were used for *in vivo* study of collagen dura substitute. All the animals were randomly assigned into three groups (i.e., experiment, control and blank control) and were feed 5 days to adapt to the environment before the surgery. The surgical procedures are shown as Figure 1. Briefly, the animals were general anesthesia with 3% pentobarbital sodium (25 mg/kg) *via* auricular vein and intramuscular injected with 100 kU penicillin, and placed in prone position. Through a 4 cm long calvarium midline incision, the skull was exposed. Behind the crest of the skull and distant around 6 mm to the midline on the right, a round opening with diameter of 12 mm was created by high-speed electric drill. The dura mater was exposed and a defect around 8 mm in diameter was made. The dura substitutes with 9 mm in diameter were applied to repair the defects in both experimental and control groups. Finally, the periosteum, subcutaneous tissue and skin were successively closed with absorbable suture. In blank control group, the defects were left without any implants but only suture. After the surgery the animals were kept in individual cages at room temperature on a 12 h light/dark cycle. Standard balanced food and water were available for freely intake. The penicillin (100 kU/kg) has been intramuscular introduced 30 min before surgery, and prolonged 3 days after the surgical procedure.

#### Observation and Harvest Procedure

One week after the operation, all the animals were daily inspected on surgical site, including the wound healing, cerebrospinal fluid (CSF) leakage and infection situation. Especially, the surgical site, middle ear cavity and accessory nasal sinuses were carefully checked as possible CSF leakage sites.

Leukocyte count in complete blood was applied at three sampling time points: before the operation, 1 week post-operation and before the sacrifice. Leukocyte count in CSF was applied with CSF was collected just before animal sacrifice. The method used for leukocyte counts was microscopic visual counting.

Immunoglobulin G (IgG) and immunoglobulin M (IgM) in serum which was collected before the operation and before the sacrifice were detected with quantitative assay kits. The testing kits used for IgG (Catalog No. KA2017) and IgM (Catalog No. KA2040) were purchased from Abnova Inc. (United States).

After the sacrifice, the tissue materials were sampled and fixed in 10% buffered formalin for 10 days, following the decalcification in decalcifying fluid. Conventional histological sample treatments and staining were applied. Briefly, representative tissue sections were placed in histopathological cassettes, and processed in the tissue processor, where the material was dehydrated gradually in series of ethyl alcohol with increasing concentrations (80–99.8%), subsequently cleared in the series of xylenes, and embedded in paraffin. The formed paraffin blocks were cut using rotary microtome (Thermo Scientific HM340E) into 5 μm paraffin tissue sections, placed on slides and stained with H&E.

The animals were sacrificed at 30, 90, and 180 days postoperatively in all three groups (experiment group, control group and blank control group) by intravenous administration of a pentobarbital overdose (50 mg/kg).

The tissue adhesion of decalcified tissue samples and tissue sections were both double-blind rated by four senior researchers.

### Grading System

A quantitative grading system was applied to score the tissue adhesion to the implants in both macroscopic and microscopic assessments, as previously described (Shackelford et al., 2002; NMPA, 2019). Macroscopic assessment of adhesion to surrounding tissue was graded from 0 = none, 1 = minimal, easy to separate, 2 = moderate, can separate without any break, 3 = multiple, difficult to separate and some tissue integrate with the graft, and 4 = extensive, hardly to separate and lots of tissue integrate with the graft. Microscopic assessment of adhesion and inflammation of the graft were graded from 0 = none, 1 = minimal (<1%), 2 = mild (1–25%), 3 = moderate (26–50%), 4 = moderately severe (51–75%), and 5 = severe.

### Statistical Analysis

All the experimental data were presented as mean ± standard deviations, and analyzed for significant by Kruskal-Wallis test, followed by Mann-Whitney U test. Data were analyzed using SPSS Statistics Software (version 17.0). Statistically significant differences were defined as having *p* < 0.05.

## Results

### Characterization of Dura Substitutes

#### Composition Analysis

**FIGURE 2:**
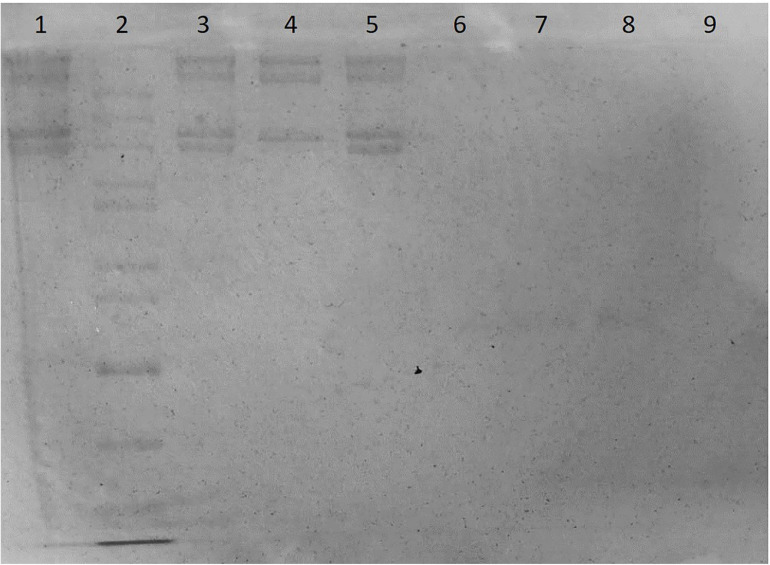
The SDS-PAGE of dura substitute (Lane 1, 3, 5: sample A, collagen solution; Lane 2: sample E, molecular weight ladder; Lane 4: sample F, control type I collagen; Lane 6: sample B, digested collagen solution; Lanes 7, 8: sample D, BSA; Lane 9: sample C, collagenase solution).

The SDS-PAGE image was shown as Figure 2, and the meanings of sample codes and lane numbers were specified in Table 1. According to the electrophoresis strips in the lanes loaded with different samples, dura substitute sample (sample A, lane 1, 3, 5) had similar molecular weights and distribution with type I collagen control (sample F, lane 4). Also, no strips were observed of collagenase digested dura substitute (sample B, lane 6) and collagenase (sample C, lane 9), which indicated the dura substitute was fully digested by collagenase. Thus, the amount of impurities in dura substitute samples was no larger than the detection limit measured with BSA. Since 20 μL of 0.01 mg/mL BSA solution was loaded as sample D (lane 7, 8), the purity of collagen dura substitute could be calculated as the following equation.

**TABLE 1:** The meaning meanings of sample codes and lane numbers.

**Sample code**	**Lane number**	**Meaning**
A	1, 3, 5	Dura substitute samples
B	6	Collagenase digested dura substitute
C	9	Collagenase
D	7, 8	BSA
E	2	Protein molecular weight ladder
F	4	Type I collagen control

$$X=\frac{V*C-L\,L_{B\,S\,A}}{V*C}\times 100\%$$

Where LL_BSA_ is the lower limit of BSA (μg), *V* is the loaded sample volume (μL) and *C* is the sample concentration (μg/μL). In this study, C was 2.64 mg/mL, V was 20 μL, and LL_BSA_ was 0.2 μg. Accordingly, the purity was 99.62% and the non-collagen impurity content was less than 0.40%.

The result of amino acid analysis was given in Table 2, which included the molar ratios of different amino acids in collagen dura substitute. Also, the amino acid compositions of bovine collagen alpha-1(I) chain (UniProtKB entry: P02453) were shown as control according to the data from UniProtKB (UniProt, 2020). It is obvious to find that the collagen dura substitute met the main composition features of type I collagen, such as around 1/3 of glycine, no cysteine and tryptophan, and close total content of proline and hydroxyproline.

**TABLE 2:** The amino acid analysis of dura substitute.

**AA**	**Molar ratio**		**AA**	**Molar ratio**		**AA**	**Molar ratio**	
	**dura**	**Bovine I α 1**		**dura**	**Bovine I α 1**		**dura**	**Bovine I α 1**
Asp	4.73%	4.26%	Val	2.26%	1.61%	Lys	1.87%	3.60%
Thr	1.79%	1.61%	Met	0.11%	0.66%	His	0.13%	0.28%
Ser	3.31%	3.60%	Ile	1.18%	0.85%	Arg	5.21%	5.02%
Glu	7.58%	7.48%	Leu	2.69%	2.08%	Pro	11.24%	22.82%
Gly	34.06%	32.77%	Tyr	0.27%	0.47%	Hyp	9.37%	0.00%
Ala	12.45%	11.65%	Phe	1.75%	1.23%	Trp	0.00%	0.00%
Cys	0.00%	0.00%						
AA, Amino Acid				Total 100.00%				

#### Structural Characterization

**FIGURE 3:**
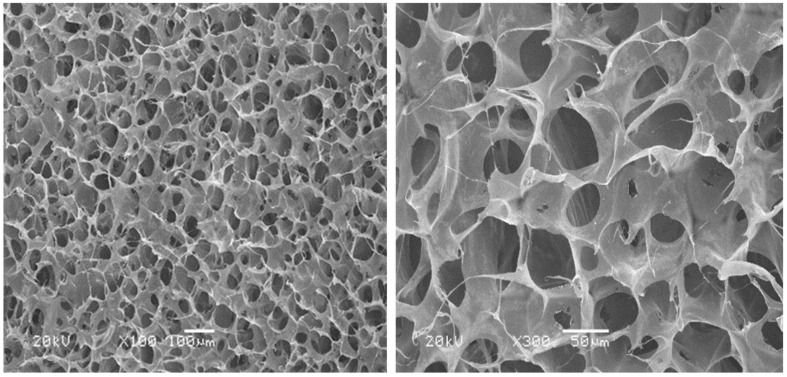
The microstructure of dura substitute.

The microstructure of the collagen dura substitute was shown in Figure 3, which demonstrated a three dimensional network structure with inter-connected pores of diameters ranged from a few to 150 μm.

### *In vivo* Animal Study

#### Gross Observation and Fluid Examination

All the animals had normal intakes and behavior post-operation. The body weight of each animal increased gradually and showed insignificant difference among groups (data not shown). The body temperature of each animal was in normal range during the observation period and showed insignificant difference among groups (data not shown).

For experiment and control groups, no irritation or inflammation, nor fluid or discharge was observed at the surgical site. More importantly, no CSF was observed postoperatively till the sacrifice of animals.

#### Inflammatory Evaluation

**FIGURE 4:**
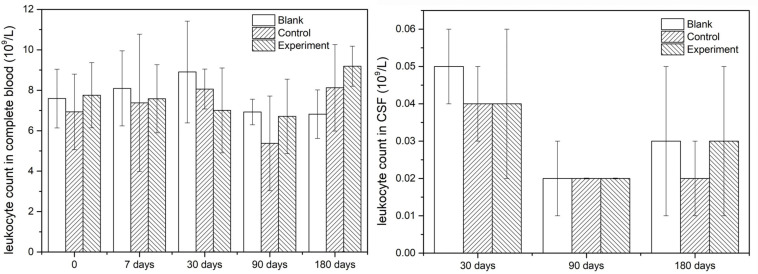
The leukocyte counts in complete blood and CSF.

Since leukocytes were considered as one of the main response results when there is an inflammatory response, the leukocyte count was detected both in complete blood and CSF in this study. The results were shown in Figure 4 and they indicated that the leukocyte counts in both complete and CSF had insignificant difference at different sampling time points, which meant no inflammatory caused by the implant or the operation.

#### Immunological Response

**FIGURE 5:**
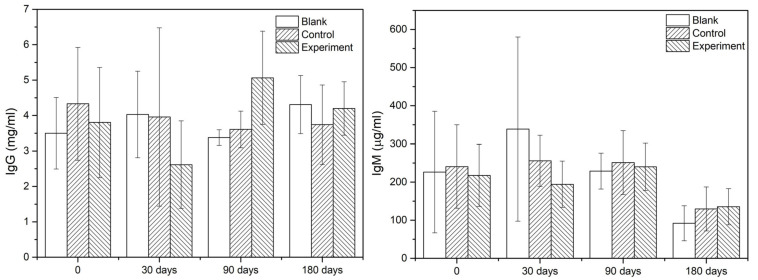
The IgG and IgM contents in serum.

As the main indicators of immunological response, the IgG and IgM contents in serum of different groups at given sampling times were showed in Figure 5. According to the results, there was no significant difference among the groups comparing the values at sampling time and those acquired before the operation. Therefore, it was reasonable to confirm that there was no early or delayed infection owing to the operation or the implant.

#### Macroscopic Examination

**FIGURE 6:**
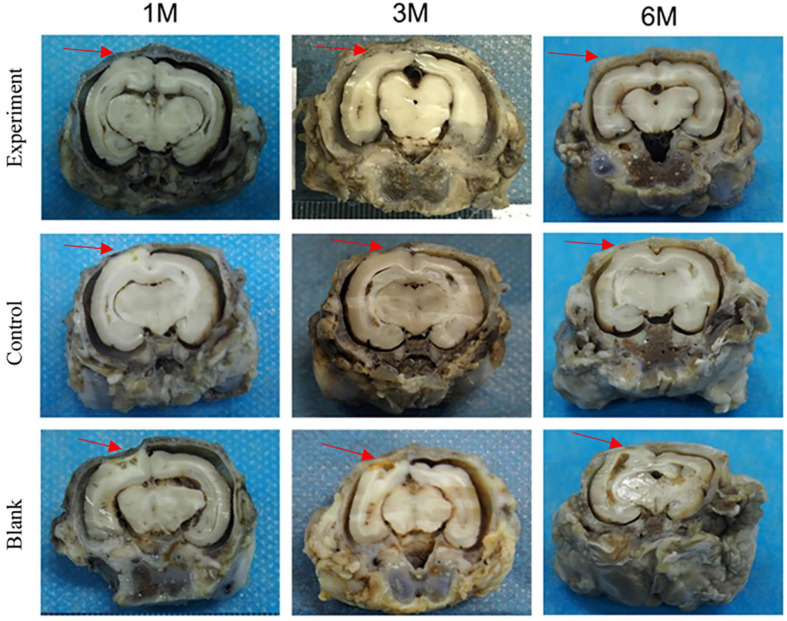
The macroscopic examination of the implants (arrow: implant position).

**FIGURE 7:**
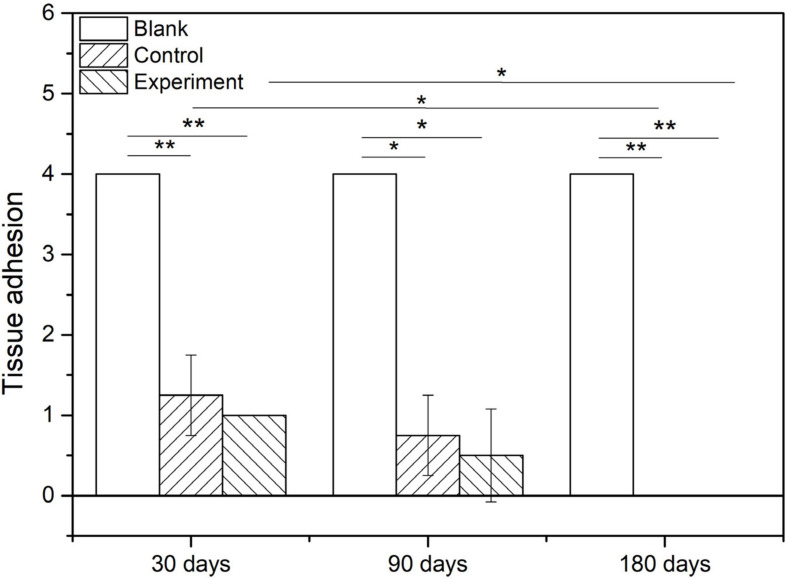
The adhesion grading of implants with tissue (*P < 0.05, **P < 0.01).

The tissue adhesion of dura substitutes is one of the critical aspects in the safety evaluation. Since there is no guidance document for tissue adhesion for animal study of dura substitutes, intraperitoneal hernia repair guidance document from NMPA and its referred evaluation standard on tissue adhesion is applied in this study. The macroscopic examination results of the implants at different sampling times were shown as Figure 6, and the adhesion grading results were given in Figure 7. For blank control group, the results showed that the adhesion of brain tissues was so severe that they were very difficult to be separated from the skull and lot of brain tissue were attached after the separation, no matter the examination time was 30, 90, or 180 days post-operation. The tissue adhesion in groups with dura substitute implantation was significant improved according to the results at the first sampling time (30 days). In both experiment and control groups, slight adhesion was found and was easy to be separated. 180 days after the implantation, there was no adhesion was observed in both experiment and control groups.

**FIGURE 8:**
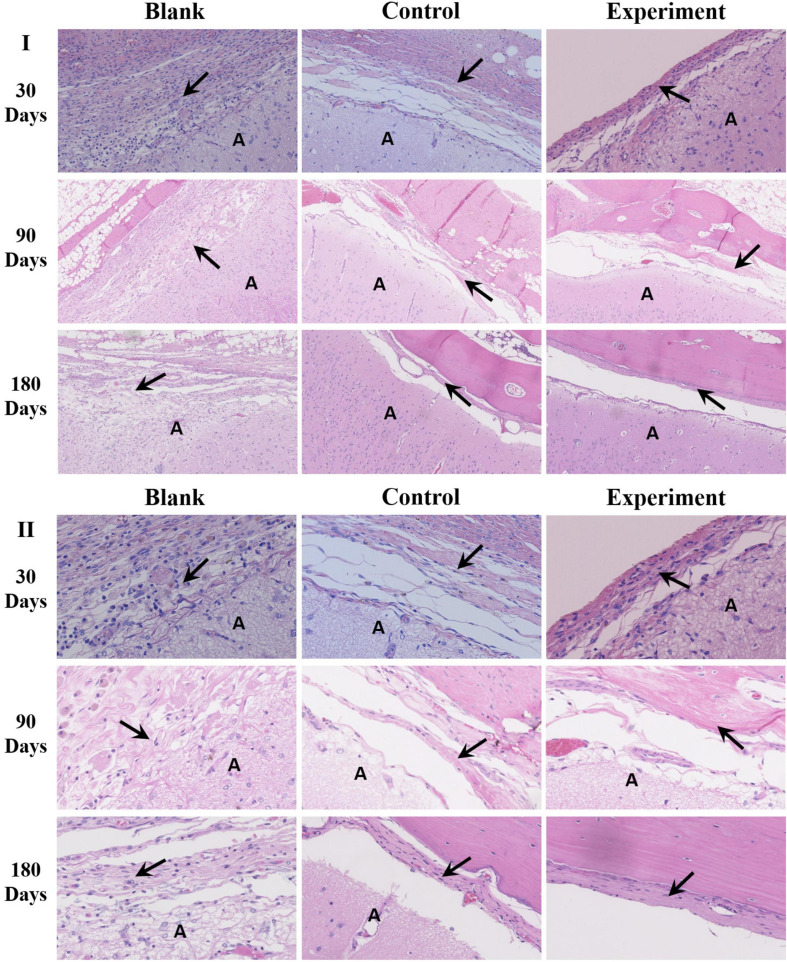
The histological observation of the implants (A: brain tissue; the arrow → pointed to the repaired tissue; I: ×100, II: ×400).

The histological results of tissue samples were shown as Figure 8. According to the microscopic images of tissues sampled at 30 days, the implants in both experiment and control groups were closely integrated with the surrounding dura tissue. At the same time, attachment and migration of fibroblasts into the implants were clearly identified while no abnormality was observed in arachnoid and brain tissues. The implants were partially degraded, and entangled with the newly formed extracellular matrix as repaired tissue. In blank control group, an obvious dura defect and disorderly fibroblastic proliferation in lateral dura was observed. Meanwhile, the arachnoid structure was incomplete and the brain tissue was shown as necrosis.

After 90 days of implantation, a large number of fibroblasts were observed in both experiment and control groups. The implanted dura substitutes were mostly degraded in both groups. The newly formed tissue with characteristic dura structure had no clear boundary with surrounding brain tissue, while the contacting sides of skull and meningeal tissue showed fine condition and no abnormality was observed in both arachnoid and brain tissue. Thus it is possible to consider that the regenerated dura had mainly replaced the implanted dura substitute. Comparatively, the blank control group exhibited obvious dura defect and loss of arachnoid structure. Also, the brain tissue was partially liquefied or necrotic, while it had visible adhesion with newly formed tissue at the edge of defect, accompanying with noticeable scar tissue.

One hundred and Eighty days after the surgery, the implants were completely degraded and the regenerated dura fully substituted the implants in both experiment and control groups. The surrounding tissues were in good conditions as previous and the skull insufficiently covered the surgery zone was mostly regenerated as well. In blank control group, dura deficiency was obvious and tissues were in similar conditions with what were observed in the same group after 90 days of the implantation.

**FIGURE 9:**
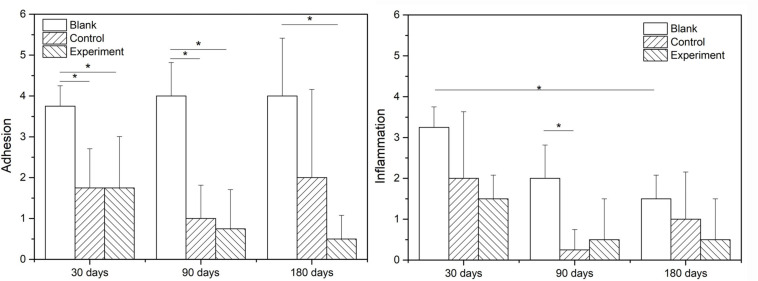
The grade scoring of adhesion and inflammation on microscopic assessment (*P < 0.05).

Figure 9 show the scoring results of histological evaluation of different groups at 30, 90, and 120 days after implantation. Thirty days after surgery, the tissue adhesion in blank control group was extensive and severe, which was significantly higher than those in experiment and control groups (*P* < 0.05). And this condition continued to 90 and 180 days after the surgery. The difference between experiment and control groups was insignificant at 30 and 90 days after the implantation, but the adhesion was insignificantly higher in control group than that in experiment group at 180 days after implantation (*P* > 0.05).

The inflammatory reactions in all groups were evaluated to be mild to moderate without obvious different at 30 days after the surgery. At 90 and 180 days after the implantation, the inflammatory cell infiltration in both experiment and control groups were reduced and significant lower than that in blank control group (*P* < 0.05), but the difference between experiment and control groups were insignificant (*P* > 0.05).

## Discussion

The composition analysis indicated the fibrous collagen prepared from bovine tendon was highly purified type I collagen (>99.5%) and the amino acid ratios met the main features of bovine collagen alpha-1(I) chain. Thus, the dura substitutes prepared by highly purified type I collagen in this study showed essential composition requirement for dura repair and potential regeneration as well. The dura substitutes possessed with excellent porous network structure which was critical to influence cell behaviors as an ideal biomimetic scaffold (Xu et al., 2017; Wang and Ao, 2019). The pores were inter-connected and the diameters ranged from a few to 150 μm, which was beneficial to the cell attachment, migration and proliferation. Also, the mass transfer, including delivery of nutrient and removal of metabolite in the dura substitute was unhindered, which was suitable for regeneration of extracellular matrix as well. Therefore, the highly purified chemical composition as well as biomimetic microstructure of the experiment dura substitute in this study is favorable niche for host fibroblasts to further regenerate damage or lost dura (Calikoglu et al., 2019).

All medical devices need to demonstrate their safety and efficacy *via* scientific evidence (Bi et al., 2020). For dura substitute devices expected to regenerate dura mater, the systematic design verification tests include, but not limited to, physical and chemical characterizations; biocompatibility evaluation per ISO 10993 standards which could include acute and chronic systemic toxicity, local tissue response, and genotoxicity tests; biological safety evaluation includes immunogenicity and infectious pathogen test of animal-derived devices. Animal studies are important to validate the *in vivo* safety as well as performance of the device per intended uses. For dura substitute devices, animal studies could provide data and evidence to prove their tissue repair and potential regeneration properties *in situ* of the damaged or lost dura mater.

*In vivo* safety and performance of collagen-based dura substitutes was investigated *via* animal studies. Multiple approaches were applied to conduct a comprehensive evaluation of the dura substitute for its ability to repair and regenerate dura mater. The gross observation, such as body weight and body temperature of the animal indicated the surgery and implant didn’t cause abnormal body reaction or pyrogen reaction. The insignificant difference of leukocyte counts in both complete blood and CSF in all three groups implied the implants caused no systematical inflammatory in short (7 days) or long (180 days) term without any encapsulation or use of antibiotic (Xu et al., 2020). Furthermore, the immunogenicity of type I collagen has been related to the end of its triple helix structure, which is composed of non-helical telopeptides. Removing these non-helical components via specially designed processes has been shown to reduce the immunogenicity of type I collagen. As a result, the immunological responses showed no infection caused by the implants, which may owe to their highly purified fibrous type I collagen that has removed non-helical telopeptide in the molecule (Lynn et al., 2004). The macroscopic examination and histological observation suggested that the collagen-based dura substitute was tissue anti-adhesion and had suitable degradation rate which was appropriate for aggregation of newly secreted extracellular matrix in a gradual and order pattern to achieve desirable regeneration effect (Wang and Ao, 2019; Ramot et al., 2020). The highly purified type I collagen fibrils and porous microstructures were important to mimic the natural tissues for triggering the native mechanisms to regenerate new host tissue, while the desirable degradation rate to match the progress of newly formed extracellular matrix was critical for dura regeneration too.

## Conclusion

The *in vivo* safety and performance of fibrous collagen dura substitutes composed of highly purified type I collagen and biomimetic porous microstructure were investigated using a rabbit model, which was feasible and reliable. Compared with the blank control, the collagen dura substitutes significantly improved the outcome of the dura repair. The collagen dura substitutes exhibited excellent *in vivo* performance in terms of anti-leaking of CSF, tissue anti-adhesion and degradability. Furthermore, the collagen dura substitutes also demonstrated dura regeneration ability during the course of animal study. In summary, the fibrous type I collagen dura substitutes in this animal study were safe and showed excellent performance for dura repair and regeneration.

## Data Availability Statement

The original contributions presented in the study are included in the article/Supplementary Material, further inquiries can be directed to the corresponding author/s.

## Ethics Statement

The animal study was reviewed and approved by the Animal Care and Use Committee of Sichuan University (IACUC-2016-R-001).

## Author Contributions

HL: conceptualization and funding acquisition. WL, XW, ZZ, and HL: methodology. XW, ZZ, YZ, and HL: validation. WL, JS, QJ, JW, YX, and ZZ: investigation. WL and HL: writing–original draft preparation. YZ and HL: supervision. All authors contributed to the article and approved the submitted version.

## Conflict of Interest

The authors declare that the research was conducted in the absence of any commercial or financial relationships that could be construed as a potential conflict of interest.
