# Supplementary material for: In vivo Evaluation of Fibrous Collagen Dura Substitutes
Source: Front Bioeng Biotechnol. 2021 Feb 18;9:628129. doi: 10.3389/fbioe.2021.628129 (PMC7930396; doi:10.3389/fbioe.2021.628129)
Supplement: Supplementary file 1 [file Table_1.DOCX]

Supplementary Table 1. The materials and their associated dura substitutes devices

| Types | Materials | Manufacturer | Device Registration No. | Device Name |
| --- | --- | --- | --- | --- |
| Synthetic | expanded polytetrafluoroethylene (ePTFE) | Bridger Biomed, Inc. | [K980548](http://www.accessdata.fda.gov/scripts/cdrh/cfdocs/cfpmn/pmn.cfm?ID=K980548) | Dura-Patch dura substitute |
|  |  | Shenzhen World Surgery Medical Device Technology Co., Ltd. | NMPA 20153131058 | Dura Substitute |
|  | expanded polytetrafluoroethylene (ePTFE) and amorphous fluoropolymer | W.L. Gore & ASSOCIATES, INC | K021477 | PRECLUDE MVP Dura Substitute |
|  |  |  | K984534 | PRECLUDE ACUSEAL Dura Substitute |
|  |  |  | K953969 | PRECLUDE Dura Substitute |
|  | Polyesterurethane (PU) | Aesculap AG | NMPA 20153130269 | Non-absorbable Dura Substitution |
|  | Polyesterurethane (PU) | Aesculap, Inc. | K960470 | Neuro-Patch |
|  | polylactic acid (PLA) | Medprin Regenerative Medical Technologies Co., Ltd. | NMPA 20173464684 | ReDura absorbable dura substitute |
|  | poly(L-lactide) PLLA and gelatin |  | NMPA 20193130590 | NeoDura dura substitute |
|  | polyglycolic acid (PGA), poly(L-lactide*-co-*ε-carpolactone) (PLCL) | Gunze Co., Ltd. | NMPA 20163463298 | Absorbable Dural Substitute |
|  | polyglactin 910 and polydioxanone | Johnson & Johnson Professionals, Inc. | K991413 | Codman Ethisorb Dura Patch |
|  | Poly(lactide-*co*-glycolide) (PLGA), Polydioxanone (PDO) | Acera Surgical, Inc. | [K172603](https://www.accessdata.fda.gov/scripts/cdrh/cfdocs/cfpmn/pmn.cfm?ID=K172603)/K161278/[K153613](https://www.accessdata.fda.gov/scripts/cdrh/cfdocs/cfpmn/pmn.cfm?ID=K153613) | Cerafix Dura Substitute |
| Nature-originated | crosslinked bovine/ porcine pericardium | Shanghai Cingular Biotech Corporation | NMPA 20173461437 | Dura Substitute |
|  |  | Beijing Balance Medical Inc. | NMPA 20173464401 | Surgical Mesh |
|  |  | Guanhao Biotech | NMPA 20173464058 | NormalGEN dura repair patch |
|  |  |  | NMPA 20173460670 | [GrandNeuro (Type B) dura repair patch](http://www.guanhaobio.com/EN/IndustryInfo.aspx?code=1001&id=182) |
|  |  | LeMaitre Vascular Inc. | K183513 | DuraSure Biologic Patch, |
|  |  | RTI Surgical, Inc. | K132850 | Bovine Pericardium Suturable Dural Graft, Tutopatch DM Graft, Tutoplast Bovine Pericardium DM |
|  |  | Bio-Vascular, Inc. | K982282/K973706/ K950956 | Dura-Guard Dural Repair Patch |
|  |  | Shelhigh, Inc. | K982101 | Shelhigh No-React Dura Shield |
|  | porcine pleural membrane | Guanhao Biotech | NMPA 20173464058 | NormalGEN dura repair patch |
|  | bacterial cellulose | Synthes Inc. | K131792 | SyntheCel Dura Repair and SyntheCel Dura Onlay |
|  |  |  | K113071 | SyntheCel Dura Replacement Devices |
|  | allograft skin | LifeCell Corporation | K061208 | LifeCell Dural Substitute Matrix |
|  | collagen membrane derived from processed bovine tendons | Beijing Bonsci Technology Co. Ltd. | NMPA 20193130272 | DuraPair |
|  |  | Beijing TianXinFu Medical Appliance Co. Ltd. | NMPA 20203130220 /20163462200 | DuraMax |
|  |  | Yantai Zhenghai Bio-tech Co., Ltd. | NMPA 20143132038 | Heal-All® Bio-membrane |
|  |  | Codman & Shurtleff, Inc. | K041518 | CODMAN DURAFORM Dural Graft Implant |
|  |  |  | K033395 | CODMAN Dural Graft Implant |
|  |  |  | K152481 | DURAFORMTM Dural Graft Implant |
|  |  | Integra Life Sciences Corporation | K092388 | DuraGen Plus Dural Regeneration Matrix-Spinal Matrix, Integra Spinal Mend Dural Regeneration Matrix |
|  |  |  | K072207 | DuraGenXs Dural regeneration matrix |
|  |  |  | K043427 | DuraGenII Dural Regeneration Matrix |
|  |  |  | K032693 | DuraGen Plus Dural Graft Matrix |
|  |  |  | K982180 | DuraGen Dural Graft Matrix |
|  |  | Collagen Matrix, Inc. | K150825 | Collagen Dural Regeneration Matrix |
|  |  |  | K061487 | DuraMatrix Collagen Dura Substitute Membranes |
|  |  |  | K040888 | DuraMatrix Collagen Dura Substitute Membrane |
|  | collagen membrane derived from processed bovine dermis | Collagen Matrix, Inc. | K141608 | Collagen Dura Membrane |
|  |  | Medtronic Neurosurgery | K161370/K063117/ K052211/K041000 | Durepair Dura Regeneration Matrix |
|  | porcine small intestinal submucosa (SIS) | Cook Biotech Incorporated | NMPA 20173466315 | Biodesign Surgisis Dural Graft |
|  |  |  | K131015 | Biodesign Onlay Dural Graft |
|  |  |  | K031850 | Durasisa Dural Substitute |
|  |  |  | K980431 | Surgisis Soft Tissue Graft |
|  | collagen derived from bovine pericardium | Aesculap, Inc. | K122791 | Lyoplant Onlay |
|  |  |  | K970851 | Lyoplant Dura Substitute |
|  | collagen and hydroxypropyl methyl cellulose (HPMC) | Integra Life Sciences Corporation | K163456/K120600 | DuraGen Secure Dural Regeneration Matrix |
